# A prospective study of complications from comprehensive abortion care services in Nepal

**DOI:** 10.1186/1471-2458-12-9

**Published:** 2012-01-05

**Authors:** Kathryn Andersen, Bela Ganatra, Sarah Stucke, Indira Basnett, Yagya B Karki, Kusum Thapa

**Affiliations:** 1 Ipas, 300 Market St., Suite 200, Chapel Hill, NC 27516, USA; 2 UNC-Chapel Hill School of Nursing, Chapel Hill, NC 27516, USA; 3 TCIC/Ipas Nepal, Family Health Division, 2nd Floor Teku, PO Box 11621, Kathmandu, Nepal; 4 Population, Health and Development (PHD) Group, Ring Road, Kathmandu, Sanepa, Nepal; 5 Paropakar Mother and Women's Hospital, Kathmandu, Nepal

**Keywords:** Induced abortion, Post-abortion complications, Nepal

## Abstract

**Background:**

In March 2002, Nepal's Parliament approved legislation to permit abortion on request up to 12 weeks of pregnancy. Between 2004 and 2007, 176 comprehensive abortion care (CAC) service sites were established in Nepal, leading to a rise in safe, legal abortions. Though monitoring systems have been developed, reporting of complications has not always been complete or accurate. The purpose of this study was to report the frequency and type of abortion complications arising from CAC procedures in different types of facilities in Nepal.

**Methods:**

A total of 7,386 CAC clients from a sample of facilities across Nepal were enrolled over a three-month period in 2008. Data collection included an initial health questionnaire at the time of abortion care and a follow-up questionnaire assessing complications, administered two weeks after the abortion procedure. A total of 7,007 women (95%) were successfully followed up. Complication rates were assessed overall and by facility type. Multivariable logistic regression was used to assess the association between experiencing a complication and client demographic and facility characteristics.

**Results:**

Among the 7,007 clients who were successfully followed, only 1.87% (n = 131) experienced signs and symptoms of complications at the two-week follow up, the most common being retained products of conception (1.37%), suspected sepsis (0.39%), offensive discharge (0.51%) and moderate bleeding (0.26%). Women receiving care at non-governmental organization (NGO) facilities were less likely to experience complications than women at government facilities, adjusting for individual and facility characteristics (AOR = 0.18; 95% CI: 0.08-0.40). Compared to women receiving CAC at 4-5 weeks gestation, women at 10-12 weeks gestation were more likely to experience complications, adjusting for individual and facility characteristics (AOR = 4.21; 95% CI: 1.38-12.82).

**Conclusions:**

The abortion complication rate in Nepali CAC facilities is low and similar to other settings; however, significant differences in complication rates were observed by facility type and gestational age. Interventions such as supportive supervision to improve providers' uterine evacuation skills and investment in equipment for infection control may lower complication rates in government facilities. In addition, there should be increased focus on early pregnancy detection and access to CAC services early in pregnancy in order to prevent complications.

## Background

Nepal's maternal mortality ratio (MMR) has declined in recent years from an estimated 539 deaths per 100,000 live births in the period 1989-1995 [1] to an estimated 281 deaths per 100,000 live births in the period 1999-2005 [2]. The maternal mortality ratio may have improved over this time period due to increased contraceptive use, a reduction in unsafe abortion and increased use of antenatal care services [3]. However, a recent study in the rural Sarlahi District of Nepal estimated that the pregnancy-related mortality ratio is over 500 deaths per 100,000 live births [4]. This study suggests that pregnancy-related mortality may be twice the current national average in more rural areas of Nepal [4].

Prior to 2002, abortion was illegal in Nepal and was considered a crime under any circumstance [5]. In this environment, women clandestinely sought abortions, and most of the procedures were unsafe, especially for poor, rural women [6]. In one hospital-based study of abortion in Nepal, deaths from abortion-related complications accounted for over half of all maternal deaths in the country [7]. Recognition that illegal abortion contributed to Nepal's high maternal mortality was instrumental in advocacy efforts to legalize abortion [8]. In March 2002, Nepal's Parliament approved legislation to permit abortion on request during the first 12 weeks of pregnancy for any reason, up to 18 weeks of pregnancy in cases of rape or incest, and up to any gestation in case of risk to the woman's life or fetal deformity.

In order to provide safe abortion services, a national safe abortion policy was formulated in 2002, and the first Comprehensive Abortion Care (CAC) unit was created at the Maternity Hospital, Kathmandu in 2004. Between 2004 and 2007, 176 CAC service sites - including government, non-governmental organizations (NGOs) and private facilities - were established in Nepal, and 71 of 75 districts had at least one government registered facility offering CAC services. CAC units offer counselling, use manual vacuum aspiration (MVA) to terminate pregnancies, provide post-CAC contraceptive methods and other reproductive health services, and identify and manage complications.

The establishment of abortion services in Nepal has resulted in a rise in safe, legal abortions. In total, 158,188 first trimester abortions were recorded in the country during the 44-month period from March 2004 to October 2007. While monitoring systems have been developed and government listed CAC sites report their cases quarterly to the Family Health Division of the Ministry of Health, reporting of complications has not always been complete or accurate. The objective of this paper is to report the frequency and type of abortion complications arising from CAC procedures in different types of facilities in Nepal.

## Methods

This study is part of a larger descriptive study of CAC facilities in Nepal conducted by Ipas, which included an assessment of CAC facilities, measurement of abortion complications and establishment of post-abortion follow-up and monitoring guidelines.

### Study design

This was a prospective study of first trimester abortion-related complications at selected CAC sites from May to July of 2008. This study is restricted to first trimester abortion complications because abortion technologies and the associated risks of complications differ between first and second trimester procedures. A cluster sampling strategy was used to select participants. First, facilities were selected using simple random sampling, and then all first trimester abortion cases at each selected facility that presented during the study period were recorded. The sampling frame consisted of 128 currently functioning, government-listed CAC sites where at least one abortion service was provided during the period from July to October 2007. Using probability sampling, 30 CAC sites were chosen from among this sampling frame. Sites included government, NGO and private facilities. The government facilities provide general health care and offer induced abortion services for fixed or reduced costs (as determined by need); the NGO and private clinics focus on reproductive health care at somewhat higher cost. Twenty-seven of the 30 CAC sites were included in the final sample; at one site, data collection did not occur because the trained CAC service provider left the country, while two other sites were excluded due to inconsistencies in personnel and non-adherence to the research protocol.

### Participants

Every woman attending the sampled CAC facilities for abortion care between May and July of 2008 was enrolled in the study. In total, 7,386 eligible patients at the 27 CAC sites received abortion care during the study period and were assessed with an initial questionnaire. Of these, 7,007 (95%), were successfully followed up either in person or by telephone interview two weeks after their uterine evacuation procedure.

### Instruments and data collection

The data on CAC complications used in this study come from two sources: a patient case history form which collected data on complications that occurred during the procedure or prior to discharge from the facility, and the complication follow-up form which collected data on complications experienced after discharge. The patient case history forms used by the facilities were modified for this study to include a more detailed medical history and specific abortion care information from admission to discharge. Complications experienced during the CAC procedure or before discharge were recorded by providers on the patient case history form. Clients were assessed with the complication follow-up form two weeks after their CAC procedure to enumerate and classify types of complications that occurred after the woman was discharged from the study facility.

Trained nurses were chosen to conduct the follow-up interviews as a way to introduce a routine system of monitoring complications and to strengthen their technical capacities with abortion-related complications. Though the nurses interviewed most clients two weeks after their procedure, some women were interviewed earlier if they returned to the facility for treatment of complications before the end of the two-week period, and some women were interviewed later due to difficulties in contacting them. All clients included in the analysis (n = 7,007) were interviewed within the 1 to 30-day period following the uterine evacuation procedure (mean duration of follow-up = 14.0 days, sd = 0.03). Details on signs and symptoms of potential complications, sites attended for treatment or referral, and treatment received were recorded in a follow-up log, and nurses advised women who were experiencing complications to return to the study facility for treatment. All women who reported complications at the two-week follow-up interview received treatment for their complications at the study facility; this allowed providers to confirm and document their complications. Nurses received monetary compensation for the additional work.

Site assessments were also conducted to evaluate the quality of care available in the sampled facilities. The site assessment consisted of a checklist of equipment, drug supply, facility procedures and personnel. Only 24 out of 27 facilities completed the site assessment; 2 NGO facilities and 1 government facility did not receive the site assessment due to...

### Analysis

The primary outcome used in this study was report of an abortion-related complication during or within two weeks of the CAC procedure. In addition, the complication type is reported for all women experiencing an abortion-related complication during the two-week follow-up period. Multiple complications are possible for each woman. Classification of complication type was based solely on provider judgment; complication reporting was not based on a standard clinical diagnostic of lab criteria or a standard definition of each complication. Note that while provider characteristics, including cadre, were not collected, relatively few midlevel providers had been certified to provide CAC at the time of this study.

The abortion complication rate was computed by dividing the number of women who had at least one complication among every 100 CAC clients. The 95% confidence interval was computed for the rate, accounting for clustering by facility. Wald tests were used to test the difference in complication rates by facility type. The complication type is reported as a frequency and the percentage of all women who experienced that complication, both overall and by facility type. Due to the small numbers for some complication types, Fisher's exact test was used to compare the differences in rates across facility types.

Clients who were and were not successfully followed over time were compared using chi-square tests for categorical variables and t-tests for continuous variables. Univariate and bivariate analysis of those who were successfully followed (n = 7,007) is presented using frequency and per cent of non-missing responses for categorical variables and mean and standard deviation for continuous variables. Multivariable logistic regression was used to assess the association between experiencing a complication and demographic and facility characteristics, accounting for clustering by facility. Statistical significance was set a priori at alpha = 0.05. Analysis was conducted using Stata 11.2.

### Ethical review

Ethical review for this study was provided by the Nepal Health Research Council for use of routine patient medical records; informed consent was obtained for standard care and not specifically for participation in the study. Privacy and confidentiality were ensured by maintaining data without personal identifiers such as name. Care was not affected by participation in the study.

## Results

### Characteristics of clients and abortion care received

A total of 7,007 women (95%) were successfully followed after their uterine evacuation procedure. There were no statistical differences for sociodemographic characteristics when comparing clients that were and were not successfully followed over time, except for rural or urban residence. Nearly 80% of urban clients were followed successfully, as compared to only 69% of rural clients (*p *< 0.001).

The distribution of the 7,007 successfully followed patients by facility type is shown in Table 1. Though only half of CAC sites were NGO facilities, 83% of patients were seen in these facilities. One third of the sampled facilities were government sites, but only 12.8% of patients were seen in government facilities. Private facilities accounted for 11% of the sampled sites but only 3.8% of patients. Most (62.7%) of the CAC clients were from the Central Development Region, the most populated region of Nepal and the location of Kathmandu valley (Table 1).

**Table 1:** Distribution of 7,007 CAC patients by facility type and region, Nepal 2008

	Facilities (n = 27)	Patients (n = 7007)	
	**n**	**n**	**(%)**

Facility Type			

Government	9	894	(12.8)

NGO	15	5849	(83.5)

Private	3	264	(3.8)

Region			

Eastern	5	532	(7.6)

Central	10	4396	(62.7)

Western	6	1049	(15.0)

Mid West	4	453	(6.5)

Far West	2	577	(8.2)

CAC patient sociodemographic information is described in Table 2. Most women were married (96.0%), lived in rural areas (57.0%), had less than secondary level education (68.6%) and were literate (74.4%). Significant differences were seen in marital status across facility types; less than 80% of private facility patients were married compared to over 96% of government and NGO facility patients (*p *< 0.05) (Table 2). In addition, there were significantly higher proportion of NGO patients were from rural areas (61.4%) compared to less than 40% of patients at government and private facilities (*p *< 0.05) (Table 2). Education was also significantly different by facility type; government and NGO facilities had a less educated patient population than private facilities where only 10.6% of patients had no education (*p *< 0.05) (Table 2). Patients of private facilities were also significantly more likely to be literate compared to patients of government and NGO facilities (*p *< 0.05) (Table 2). The mean patient age was 27 years (sd = 5.8, range: 14-50), and the mean gravidity and parity were 3.2 (sd = 1.63) and 1.8 (sd = 1.36), respectively. Patients at private facilities were younger (mean = 26, sd = 6.28) and had lower gravidity and parity compared to patients at government and NGO facilities (Table 2). According to the national CAC protocol, providers verified gestational age before performing the abortion procedure using last menstrual period (LMP) and clinical examination; only 25 women received an ultrasound. The mean pregnancy duration was 7.1 weeks (sd = 1.64) based on LMP and 7.4 weeks (sd = 1.75) based on clinical exam.

**Table 2:** Sociodemographic and uterine evacuation procedure characteristics of 7,007 CAC patients, Nepal 2008

	Overall (n=7,007)		Government (n=894)		NGO (n=5,849)		Private (n=264)	
	**n**	**(%)**	**n**	**(%)**	**n**	**(%)**	**n**	**(%)**

Married	6728	(96.0)	863	(96.5)	5655	(96.7)	210	(79.6)

Rural residence	3997	(57.0)	325	(36.4)	3592	(61.4)	80	(30.3)

Literate	5215	(74.4)	615	(68.8)	4364	(74.6)	236	(89.4)

Education								

None	1792	(25.6)	279	(31.2)	1485	(25.4)	28	(10.6)

Primary or some secondary	3010	(43.0)	244	(27.3)	2657	(45.4)	109	(41.3)

School Leaving Certificate or higher	2205	(31.5)	371	(41.5)	1707	(29.2)	127	(48.1)

	**Mean**	**(sd)**	**Mean**	**(sd)**	**Mean**	**(sd)**	**Mean**	**(sd)**

Age, years	26.9	(5.80)	27.2	(5.68)	26.9	(5.79)	26.0	(6.28)

Gravidity	3.2	(1.63)	3.1	(1.40)	3.3	(1.66)	2.5	(1.50)

Parity	1.8	(1.36)	1.9	(1.28)	1.9	(1.37)	1.2	(1.27)

Pregnancy duration, weeks								

Last menstrual period (n=6539)	7.1	(1.64)	7.3	(1.46)	7.0	(1.66)	7.3	(1.68)

Clinical exam (n = 6307)	7.4	(1.75)	7.6	(1.54)	7.4	(1.78)	7.3	(1.58)

Ultrasound (n = 25)	7.9	(1.96)	7.7	(1.89)	8.7	(2.52)	8.5	(3.53)

	**n**	**(%)**	**n**	**(%)**	**n**	**(%)**	**n**	**(%)**

Prophylactic administration of antibiotics	5277	(75.3)	809	(90.5)	4217	(72.1)	251	(95.1)

Post-abortion contraceptive method								

No method accepted	1328	(19.0)	134	(15.0)	1180	(20.2)	14	(5.3)

Condom	2715	(38.8)	103	(11.5)	2555	(43.7)	57	(21.6)

Depo Provera	1461	(20.9)	343	(38.4)	1022	(17.5)	96	(36.4)

Pills	1035	(14.8)	175	(19.6)	790	(13.5)	70	(26.5)

Norplant	136	(1.9)	11	(1.2)	124	(2.1)	1	(0.4)

IUD/IUCD	237	(3.4)	76	(8.5)	140	(2.4)	21	(8.0)

Sterilization	95	(1.4)	52	(5.8)	38	(0.7)	5	(1.9)

All 7,007 patients received first trimester CAC services using MVA. Almost all patients (99%) received some type of pain management (paracervical block with 1% xylocaine and/or oral analgesics), and cervical priming was conducted for 1% of clients (results not shown). Infection prevention practices varied by facility type. Only 63% of government facilities and 67% of private facilities had adequate equipment for infection control, including a heat source for high-level disinfection of instruments and supplies such as antiseptic solution and detergent powder, compared to 92% of NGO facilities (results not shown). Government and private facilities administered antibiotics prophylactically to over 90% of patients, while only 72% of patients at NGO facilities received antibiotics (*p *< 0.05) (Table 2). The mean length of stay from procedure to discharge among all CAC clients was 26 min (sd = 20.4) and ranged from 17 min in government facilities to 42 min in private facilities (results not shown). More than 99% of clients received contraceptive counselling (results not shown), and 81% left with a family planning method (Table 2). Among women who accepted family planning, the most popular methods included condoms (38.8%), Depo Provera (20.9%) and oral contraceptives (14.8%) (Table 2). Provision of family planning methods differed by facility type; while nearly one-fifth of women left NGO facilities without a method, only 15% of women at government facilities and 5% of women at private facilities left without a method (Table 2).

### Abortion complications

Among the 7,386 patients receiving care at participating CAC centers, 17 experienced signs and symptoms indicative of complications during or immediately after the induced abortion procedure. This resulted in a complication rate of 0.23 per 100 patients (95% CI: 0.05-0.41) at the time of the procedure. The rates varied by facility type (*p *< 0.05); government facilities reported a complication rate of 0.42 per 100 patients (95% CI: 0.03-0.80) while NGOs reported a complication rate of 0.21 per 100 patients (95% CI: 0.005-0.42). Private facilities did not report any complications during CAC procedures.

Additionally, 131 women experienced signs and symptoms of complications during the follow-up period, yielding a complication rate of 1.87 per 100 women (95% CI: 0.51-3.23). The number and percentage of patients reporting each type of complication are listed in Table 3 for the overall sample and by facility type. The most frequently reported post-procedural complications included retained products of conception (RPOC) (1.37%), offensive discharge (< 1%), moderate bleeding (< 1%) and suspected sepsis (< 1%) (Table 3). There were no reported cervical tears/lacerations, uterine perforations, or missed ectopic pregnancies. Cases of RPOC were explored in greater detail, and it was found that these cases were clustered in fewer than half of the providers (results not shown). Twelve NGO providers (34%), 24 government providers (41%) and only 1 private provider (10%) had at least one patient with RPOC (results not shown).

**Table 3:** Signs and symptoms of complications at time of follow-up among 7,007 CAC clients, overall and by complication type, Nepal 2008

	Overall (n=7,007)		Government (n=894)		NGO (n=5,849)		Private (n=264)	
	**n**	**(%)**	**n**	**(%)**	**n**	**(%)**	**n**	**(%)**

Total complications*	131	(1.87)	53	(5.93)	70	(1.20)	8	(3.03)

*Complication Type**^†^***								

Retained products of conception*	96	(1.37)	38	(4.25)	55	(0.94)	3	(1.14)

Offensive discharge*	36	(0.51)	8	(0.89)	24	(0.41)	4	(1.52)

Bleeding								

Moderate*	18	(0.26)	5	(0.56)	10	(0.17)	3	(1.14)

Severe with clots*	4	(0.06)	4	(0.45)	0	(0)	0	(0)

Suspected sepsis* ^‡^	17	(0.24)	5	(0.56)	8	(0.14)	4	(1.52)

Fever*	11	(0.16)	2	(0.22)	7	(0.12)	2	(0.76)

Uterine atony*	5	(0.07)	4	(0.45)	1	(0.02)	0	(0)

Failed abortion*	4	(0.06)	4	(0.45)	0	(0)	0	(0)

Hematometra	3	(0.04)	2	(0.22)	1	(0.02)	0	(0)

Tender uterus	3	(0.04)	2	(0.22)	1	(0.02)	0	(0)

Localized peritonitis	3	(0.04)	0	(0)	2	(0.03)	1	(0.38)

Hypovolaemic shock	1	(0.01)	1	(0.11)	0	(0)	0	(0)

Tip of cannula inside uterus	1	(0.01)	0	(0)	1	(0.02)	0	(0)

Offensive products	1	(0.01)	0	(0)	1	(0.02)	0	(0)

*p < 0.05

**^† ^**The sum of complications indications by type is greater than 131 because women could experience more than one indication of their complication

^‡ ^Suspected sepsis is reported because it was indicated by providers; however clinical signs and symptoms suggest that these women were experiencing signs of infection, rather than true sepsis.

The complication rate and types of complication during follow-up were also compared across facility type (Table 3). Patients at government facilities experienced the highest complication rate (5.93 per 100 patients; 95% CI: 4.9-7.0), followed by private facilities (3.03 per 100 patients; 95% CI: -2.0-8.1) and NGO facilities (1.2 per 100 patients; 95% CI: 0.2-2.2) (Table 3). Some complication types, such as RPOC, severe bleeding, uterine atony and failed abortion, were significantly more common in government facilities and others, such as offensive discharge, suspected sepsis and fever, were more common in private facilities (*p *< 0.05) (Table 3). The complication rate was also assessed by residence to determine whether the lower rate of follow-up for rural patients affected the complication rate; rural patients had a significantly lower total complication rate (1.55 per 100 women) than urban patients (2.29 per 100 women) (*p *< 0.05) (results not shown).

In total, 143 of the 7,007 patients with successful follow-up contact had a documented complication either during the procedure, during the follow-up period, or both, for an overall complication rate of 2.0 per 100 procedures (95% CI: 0.63-3.45) (results not shown). Significant differences in overall complication rates are observed by facility type; NGO facilities have a complication rate of 1.4 per 100 procedures (95% CI: 0.3-2.4), compared to 6.0 per 100 procedures (95% CI: 4.9-7.1) in government facilities (p < 0.05) (results not shown). Women receiving care at NGO facilities have one fifth the odds of experiencing a complication compared to those treated at government facilities (unadjusted OR = 0.22; 95% CI: 0.15-0.31) (results not shown). Table 4 presents results from multivariable logistic regression of the association between experience of a complication and demographic and facility characteristics. After adjusting for facility and patient characteristics, women receiving care at NGO facilities had significantly lower odds of experiencing a complication than women receiving abortion care from government facilities (AOR = 0.18; 95% CI: 0.08-0.40) (Table 4). In addition, women who presented for CAC services at higher gestational ages had higher odds of experiencing a complication than women who presented at 4-5 weeks gestation (Table 4). At 6-7 weeks gestation, women had 3 times higher odds of experiencing a complication (95% CI: 1.02-8.65), and at 10-12 weeks gestation, women had 4 times higher odds of experiencing a complication (95% CI: 1.38-12.82) (Table 4).

**Table 4:** Abortion-related complications* by select facility and patient characteristics among 7,007 CAC clients, Nepal 2008

	Complications			
	**n**	**(%)**	**AOR**	**95% CI**

*Facility Characteristics*				

Facility Type				

Government	54	(6.0)	1.00	

NGO	80	(1.4)	0.18	(0.08-0.40)

Private	8	(3.0)	0.41	(0.08-2.17)

Development Region				

Central	101	(2.3)	1.00	

East	5	(0.9)	0.62	(0.05-7.48)

West	23	(2.2)	1.79	(0.66-4.86)

Mid West	10	(2.2)	0.81	(0.45-1.45)

Far West	3	(0.5)	0.72	(0.23-2.24)

Clinic Location				

Urban	131	(2.3)	1.00	

Rural	11	(0.8)	0.51	(0.22-1.18)

*Patient Characteristics*				

Age				

15-19	11	(2.0)	1.00	

20-29	85	(2.0)	0.78	(0.35-1.75)

30-50	46	(2.0)	0.82	(0.35-1.92)

Residence				

Urban	73	(2.4)	1.00	

Rural	69	(1.7)	1.04	(0.66-1.64)

Gravidity				

1	24	(2.2)	1.00	

2	28	(2.1)	0.87	(0.44-1.73)

3	38	(2.0)	0.83	(0.44-1.57)

4 or more	52	(2.0)	0.94	(0.40-2.23)

Gestational Age, weeks				

4-5	4	(0.6)	1.00	

6-7	74	(2.0)	2.98	(1.02-8.65)

8-9	32	(2.1)	2.59	(0.70-9.59)

10-12	19	(2.8)	4.21	(1.38-12.82)

*Includes complications that occurred during the procedure and those that were reported at the two-week follow up interview.

### Management of abortion complications

All of the 131 clients with signs and symptoms of complications received treatment at the facility where they had their initial abortion procedure. Among the 96 women with reported RPOC, 89 (93%) underwent re-evacuation, and of those women, 11 received oxytocin/methargin and six received IV antibiotics. All six women who received IV antibiotics for treatment of RPOC attended government facilities; women who were treated in NGO and private facilities did not receive IV antibiotic treatment (results not shown). Among the five women with reported atony, four were treated by administration of oxytocin/methargin, according to the complication management protocol (results not shown).

Although antibiotic administration is included in every protocol for management of abortion complications post-procedure, the majority of the clients presenting with complications in this study did not receive antibiotics. Only 36 (27%) of the 131 women with complications received antibiotics-including 5 (29%) of the 17 cases of suspected sepsis and 15 (68%) of the 22 cases of moderate or severe bleeding (results not shown). A higher proportion of women treated in government facilities received antibiotic treatment for complications (37.7%) compared to NGO (20%) and private facilities (25%) (results not shown).

## Discussion

This study found that complications arising from safe abortion services in Nepal were few (2% of all cases), with the most common complications being RPOC (1.37%), suspected sepsis, offensive discharge and moderate bleeding, all at less than 1%. The complication rate is similar to that found in other settings in Asia, including India and Vietnam and in sub-Saharan Africa, including Kenya, South Africa [9-11]. This suggests that, overall, CAC services in Nepal are being provided safely. However, differences in complication rates across facility types highlight areas for improvement in the prevention and management of abortion complications.

Though the overall complication rate was similar to other settings in developing countries, the rate of RPOC (1.37%) was somewhat higher than the expected range of 0.5-1.2% of induced abortion cases [10,12]. The finding that patients with RPOC are concentrated among fewer than half of the providers indicates that training to improve uterine evacuation skills may need to be focused on this subset of providers. Specifically, training may need to focus on examination of the products of conception immediately after the procedure to determine whether all products have been removed. Other options include use of transvaginal ultrasound after the CAC procedure would also aid in determining whether products of conception remain [13]; however investment in this technology would be most cost-effective in facilities with a high caseload and is not generally feasible in a low resource setting, such as Nepal. In addition, facilities may reduce their cases of RPOC through administration of oxytocics such as methargin after the CAC procedure to aid in the expulsion of small fragments of products of conception not removed during the procedure [12]. Ideally, further training on inspecting products of conception immediately following MVA would reduce the rate of RPOC to expected levels.

Although most women sought abortion care early (66% up to and including seven weeks), those who sought CAC services at 10-12 weeks gestation were four times more likely to have a complication than those who sought care at 4-5 weeks gestation. More effort should be directed towards early pregnancy detection and increased access to services early in pregnancy in order to increase the likelihood of a safe abortion and reduce the risk of complications [14].

Despite the low rate of complications overall, disparities in complication rates were observed by facility type. Women who received care at NGO facilities had one-fifth the odds of experiencing a complication compared to women who received care at government facilities. Providers in NGO facilities have a higher abortion caseload compared to providers in government facilities, and it is likely that the lower complication rate in NGO facilities is related to providers' greater experience performing CAC procedures. Providers in facilities with a low caseload may benefit from interventions such as supportive supervision, which aim to improve providers' uterine evacuation skills through modelling of correct practices [15]. However, higher caseload may also be associated with lower quality of care if providers do not have sufficient time to dedicate to each patient; further research is needed to assess the effect of caseload on quality of care. It is also likely that NGO facilities have lower complication rates due to a better infection control environment compared to government facilities; over 90% of NGO facilities were found to have all of the equipment needed for infection prevention procedures compared to only 63% of government facilities. While complications such as RPOC are related to provider skill, complications such as suspected sepsis are related to infection control procedures; both types of complications were more common in government facilities than NGO facilities. Interventions such as supportive supervision to improve providers' uterine evacuation skills and investment in equipment for infection control may lower both types of complications in government facilities.

Though government facilities had higher complication rates than NGO facilities, they were also more likely to manage abortion complications according to the protocol. Antibiotic administration is a part of Nepal's protocol for managing *all *abortion-related complications [16], but this study shows that this practice is not universal. Antibiotic administration is especially important to reduce the morbidity associated with suspected sepsis, but only 29% of women who presented with suspected sepsis received antibiotics. Additional provider training efforts should emphasize the importance of universal use of antibiotics for treatment of complications [17].

This study had several limitations. Though 95% of clients were followed up, the follow-up rate was significantly lower for rural women, who may have been unable to return to the facility for their follow-up interview due to the expense of transportation and/or may not have had access to a telephone so that the follow-up interview could be conducted, compared to urban women who would be expected to have greater access, which may bias the results. Since complication rates would be expected to be equivalent between rural and urban areas, the finding that the complication rate was lower among rural women may suggest that some complications were missed among rural women who were not followed up. As a result, our estimate of the overall complication rate may be an underestimate. Another limitation is that classification of complications was based on provider judgment rather than standardized definitions of complications, and as a result, the definition of each complication may not be consistent across providers. This may be a particular problem with our estimate of the number of cases of suspected sepsis due to a lack of systematic documentation of clinical findings across participating sites.

## Conclusions

While the overall rate of CAC complications in Nepal is low and similar to the rates found in studies from other parts of Asia and sub-Saharan Africa [10-12], of concern is the finding that complication rates varied significantly by facility type. Women who received care at NGO facilities had one-fifth the odds of experiencing a complication compared to women who received care at government facilities. Interventions such as supportive supervision to improve providers' uterine evacuation skills and investment in equipment for infection control may lower complication rates in government facilities. RPOC represented the greatest burden of complications, and improved practices such as routine examination of the products of conception immediately following the procedure are viable strategies to lower rates of RPOC. In addition, this study found significantly higher complication rates at higher gestational ages. This suggests that there should be increased focus on early pregnancy detection and access to CAC services early in pregnancy in order to prevent complications. Deficits also existed in the management of abortion complications; training should emphasize the importance of universal use of antibiotics for treatment of abortion complications.

## Competing interests

The authors declare that they have no competing interests.

## Authors' contributions

KA participated in study design, and led the analysis and manuscript writing process. BG conceived the study, led study design, and participated in analysis and manuscript development. SS supported study logistics and participated in interpretation of findings and manuscript development. IB participated in study conception, design, interpretation of findings and dissemination activities. YK managed data collection and participated in analysis and manuscript development. KT participated in study design and interpretation of findings. All authors read and approved the final manuscript.

## Pre-publication history

The pre-publication history for this paper can be accessed here:

http://www.biomedcentral.com/1471-2458/12/9/prepub
